# Use of Emerging Technologies and Non-*Saccharomyces* spp. for Tailoring the Composition of Yeast Derivatives: Effect on White Wine Aging

**DOI:** 10.3390/foods14040652

**Published:** 2025-02-14

**Authors:** Sabrina Voce, Anna Bortolini, Lara Tat, Andrea Natolino, Piergiorgio Comuzzo

**Affiliations:** Dipartimento di Scienze Agroalimentari Ambientali e Animali, Università degli Studi di Udine, Via Sondrio 2/A, 33100 Udine, Italy; bortolini.anna@spes.uniud.it (A.B.); lara.tat@uniud.it (L.T.); andrea.natolino@uniud.it (A.N.); piergiorgio.comuzzo@uniud.it (P.C.)

**Keywords:** yeast derivatives, non-*Saccharomyces* spp., high hydrostatic pressure, ultrasounds, polysaccharides, antioxidants, aroma compounds, wine aging

## Abstract

Yeast derivatives are additives commonly used in winemaking for different purposes. Their manufacturing process is not well standardized, being mostly based on thermal inactivation and enzyme-induced lysis; furthermore, the main strain currently authorized for their production belongs to *Saccharomyces* spp. In this study, *Saccharomyces cerevisiae* and *Torulaspora delbrueckii* were used as starting microorganisms, whereas ultrasounds and high hydrostatic pressure were performed to induce autolysis, with the aim to evaluate the possibility to use different strains and emerging technologies as alternatives to the traditional methods to produce yeast derivatives. The chemical composition of the products obtained as well as the volatile profile of wines aged on yeast derivatives were mostly affected by the treatments performed during the manufacturing process. *T. delbrueckii* showed a good aptitude as starting microorganism for producing derivatives, whereas emerging, non-thermal technologies could replace the traditional methods for inducing autolysis, allowing to obtain products with enhanced content of polysaccharides (up to 178 mg/g) and antioxidant compounds (up to 9 µmol/g), and with low odor impact. The possibility to manage the chemical composition of yeast derivatives for specific winemaking purposes may thus be possible, by using specific starting microorganism and by applying the most suitable treatment to induce autolysis.

## 1. Introduction

Yeast derivatives (YDs) are additives commonly used in winemaking as fermentation enhancers, during the rehydration phase of active dry yeasts; the release of amino acids, vitamins and sterols allows a better adaptation of the starter culture to the medium and reduces the risk of stuck fermentation occurrence [1]. YDs are also added as wine quality enhancers; by simulating aging on lees (AOL), an improvement of wine quality and stability in shorter time have been reported [2,3], thus possibly reducing the conventional period of AOL.

Among these products, mannoproteins have been widely studied since they represent the most common derivatives used in winemaking; they are well-known for their positive effect towards protein [4] and tartaric stabilization [5], color stability and wine astringency [3,6], and protection against oxidations [4,7]. Furthermore, an impact on wine aroma profile and sensory perception has been also observed [8], mainly due to the interaction with wine aroma compounds [9,10]. This modulation effect is generally dependent on the type and composition of yeast derivative [11], pH, temperature, type of aroma compounds [10,12] and contact time [3].

Concerning the production of yeast derivatives, the only yeast authorized by the International Organization of Vine and Wine (OIV) belongs to *Saccharomyces* spp., whereas non-*Saccharomyces* spp. strains may be only used for producing derivatives with a guaranteed level of glutathione [13]. The latter yeast strains have gained increasing interest during the last decades in relation to their use during alcoholic fermentation, thanks to their contribution in improving wine aroma profile [14,15] and color stability [16]. However, their most interesting feature is their ability to release polysaccharides [17] and glutathione [18] in a higher amount compared to *Saccharomyces* spp.; this may favor an improvement of wine quality and stability in a shorter time or a reduction of the conventional period of AOL [19,20]. Furthermore, extracts obtained by *Schizosaccharomyces japonicus* seem to be suitable for protein stabilization [21], and the use of *Hanseniaspora uvarum* has given interesting results for producing yeast autolysates with enhanced antioxidant properties and polysaccharides content [22].

Regarding to the manufacturing process of YDs, the most common methods used comprise, on one hand, thermal treatment for extracting mannoproteins [23] and, on the other hand, enzyme-assisted lysis and induced autolysis for producing yeast autolysates [11]. The high temperature reached during the manufacturing process, as well as pH and autolysis promoter [24], temperature [25], type of enzyme, dosage [26] and enzyme-to-substrate ratio [27], may strongly affect the chemical composition and the volatile profile of YDs.

In this regard, interesting results have been reported concerning the application of emerging technologies for processing fermentation lees and for producing YDs. The use of ultrasounds (US) has allowed a faster release of proteins and polysaccharides during wine AOL [28], and the addition of lees previously treated by high hydrostatic pressure (HHP) has led to a better color evolution, higher protection against oxidation and better aroma and sensory profile of white wine during aging [29]. The induction of autolysis in *Saccharomyces* spp. by pulsed electric fields (PEFs) may be useful for mannoproteins’ extraction [30], whereas high-pressure treatments have given promising results for yeast derivatives production [31,32]. However, to the authors’ knowledge, little is reported about the simultaneous use of different strains and methods for the production of YDs, also considering the impact on the chemical composition and volatile profile of the products obtained. In addition, few data are available about the effect on the color evolution and aroma profile of a white wine aged on YDs, obtained starting from different yeast strains and by means different technologies, also in relation to the contact time.

Considering the above, this work aimed at evaluating the impact of both yeast strains and methods for inducing autolysis on the chemical composition and volatile of yeast derivatives, to obtain innovative products with enhanced antioxidant properties, polysaccharides content and low odor impact.

*Saccharomyces cerevisiae* and *Torulaspora delbrueckii* were used as starting microorganisms; thermolysis and enzyme addition were used as traditional methods, whereas ultrasounds (US) and high hydrostatic pressure (HHP) were tested as emerging, non-thermal technologies for producing YDs. The YDs thus obtained were added to a white wine to evaluate their effect on its chemical composition and volatile profile, after two and six months of aging.

## 2. Materials and Methods

### 2.1. Chemicals

Sodium hydroxide, N-acetyl-L-cysteine, *o*-phthaldialdehyde (OPA), isoleucine, ethanol (96 % *v*/*v*), mannan from *S. cerevisiae*, sodium citrate, trifluoroacetic acid, L-glutathione reduced, L-cysteine, *p*-benzoquinone, 3-mercaptopropionic acid, bovine serum albumin (BSA) fraction V, Folin–Ciocalteu phenol reagent, 2,2-diphenyil-picrylhydrazyl (DPPH) and 4-(dimethylamino)-cinnamaldehyde (DAC) were purchased from Sigma Aldrich (St. Louis, MO, USA). Absolute ethanol and methanol (HPLC grade) were purchased by VWR Chemicals (Milan, Italy). Tartaric acid, boric acid, hydrochloric acid (37 % *v*/*v*), copper sulfate pentahydrate, sodium tartrate and sodium carbonate were purchased from Carlo Erba Reagents (Milan, Italy). MilliQ water was produced by a MilliQ Advantage A10 apparatus (Merck Millipore, Billerica, MA, USA) and microfiltered at 0.22 μm before use.

### 2.2. Yeast Strains and YDs Production

The commercial active dry yeast (ADY) preparations of *Saccharomyces cerevisiae* (strain VP5, Enologica Vason S.p.A, Verona, Italy) and *Torulaspora delbrueckii* (Prelude, Chr. Hansen, Hørsholm, Denmark) were used as starting microorganisms. Yeast suspensions were prepared by rehydrating ADY in 10 volumes of warm water, then subjected to different treatment for inducing autolysis. Thermal treatment (hereafter referred to as THERM) was performed in autoclave at 121 °C for 2 h; for enzyme addition (hereafter referred to as ENZ), a commercial preparation with β-glucanase activity (Mannozym, Enologica Vason S.p.A, Verona, Italy) was added at a concentration of 5% (*w*/*v*). These two treatments were considered as traditional methods for producing yeast derivatives. Ultrasounds treatment (hereafter referred to as US) was carried out using a Sonopuls ultrasonic homogenizer (Bandelin electronic GmbH & Co. KG, Berlin, Germany) equipped with titanium probe (13 mm diameter) (Sonotrode S26d14, Hielscher Ultrasonics GmbH, Teltow, Germany). The depth of the probe was set at 10 mm from the bottom of a 150 mL beaker containing 100 mL of cell suspension; during the treatment, samples were put in an ice bath to avoid excessive heating. The treatment was performed in continuous pulse mode, at constant frequency of 20 kHz, amplitude of 90%, sonication time of 10 min, nominal power of 200 W, adsorbed energy density of 104.68 J/mL, five cycles of sonication treatment and temperature range of 20–45 °C. Lastly, 100 mL of yeast suspensions were transferred in polyamide/polyethylene sterile bags, then subjected to high hydrostatic pressure (hereafter referred to as HHP). The treatment was carried out by an external facility (HPP Italia S.r.l., Traversetolo, PR, Italy); the processing system used was an Avure HHP machine, model AV-10 (AVURE Technologies, Erlanger, KY, USA), and the processing parameters were 400 MPa for 8 min at 30 °C. Except for THERM, the pH of suspensions was adjusted to 5.00 and they were incubated at 45 °C for 24 h to allow yeast autolysis by favoring the activity of exogenous/endogenous enzymes. After incubation, the suspensions were heated at 80 °C for 2 min to inactivate the enzymes and to stop autolysis. The suspensions were then freeze-dried and characterized in model solution (ethanol 12% *v*/*v*, tartaric acid 33 mM, pH 3.2) as reported below. All the treatments were performed in triplicates.

### 2.3. Wine Aging with YDs Supplementation

A white wine supplied by a local producer (Friuli Venezia Giulia, Italy) was used to assess the effect of the addition of YDs on color evolution and volatile profile during aging. The initial chemical composition of the white wine was as follows: pH 3.14, total acidity 4.95 g/L (as tartaric acid), free sulfur dioxide 24 mg/L, color (absorbance 420 nm) 0.06, browning test (POM-test) 117%, total polyphenols index (TPI) 5.8 and flavanols 7.1 mg/L. Doses of 0.3 g/L of yeast derivative powders were added to wines, bottled in 100 mL white glass bottles and manually sealed with crown caps after nitrogen blowing in the headspace; for aging, the wine samples were stored at a temperature of 20 + 1 °C and homogenized weekly, to simulate *bâtonnage*. Three independent replicates were prepared, and the chemical characterization of wines was carried out after two and six months after the addition of YDs. Wines with 50 mg/L of sulfur dioxide added were used as reference (hereafter referred to as CON). Except for wine aroma compounds, the samples were centrifuged (3000 rpm for 15 min) and subjected to the chemical evaluations, as described below.

### 2.4. Amino Acids and Proteins

The amount of amino acids and proteins was determined on the soluble fraction of YDs after resuspension (1% *w*/*v*) in model solution and centrifugation (3000 rpm for 10 min). Free amino acids were determined by OPA derivatization as reported by Dukes and Butzke [33]. Two buffer solutions were prepared: OPA buffer (0.671 g/L of OPA, 3.837 g/L of sodium hydroxide, 8.468 g/L of boric acid, 0.816 g/L of N-acetyl-L-cysteine) and reference buffer; the latter was prepared similarly to OPA buffer without the addition of the derivatizing agent. After centrifugation, 50 µL of samples were transferred into two different 10 mm optical path length quartz cuvettes, one containing OPA buffer and one containing reference buffer. After 10 min, the absorbance at 335 nm was measured against water, and the difference in absorbance (abs OPA−abs Ref) was used for calculating the concentration of free amino acids, according to a calibration curve prepared with standard solution of isoleucine (0–10 mM).

The concentration of soluble proteins was evaluated by Lowry assay as reported by Comuzzo et al. [7]. Briefly, after centrifugation, 400 µL of the supernatant was transferred into 10 mm optical path length glass cuvette. A mixture of 2% *w*/*v* sodium carbonate in 0.1 M sodium hydroxide solution (1.96 mL), 1% *w*/*v* copper sulfate pentahydrate (20 µL) and 2% *w*/*v* sodium tartrate (20 µL) was added, and the sample was carefully homogenized. After 10 min at room temperature, 200 µL of Folin–Ciocalteu reagent was introduced and the content of the cuvette was further mixed. After an additional 30 min, the absorbance of the sample was measured at 750 nm, against a blank obtained by replacing the samples with 400 µL of MilliQ grade water. The concentrations proteins were calculated according to a calibration curve prepared with standard solution of bovine serum albumin (0–1000 mg/L), respectively.

### 2.5. Soluble Polysaccharides and Insoluble Solids

Total soluble polysaccharides were determined in both YDs powders and wine samples after two and six months of aging. In the case of YDs, the powders were firstly resuspended at 10% (*w*/*v*) in model solution. After centrifugation (3000 rpm, 10 min), 5 mL of supernatant (from YDs suspensions and wine samples) were added to 5 volumes of ethanol (96%) and kept at 4 °C for 24 h, then the content of polysaccharides was determined by SE-HPLC, as reported by Voce et al. [22]. Briefly, the precipitated pellets were washed twine, then resuspended in MilliQ grade water and filtered on 0.22 µm cellulose acetate membrane before injection. SE-HPLC separation was achieved using a binary pump Model LC 250 (Perkin-Elmer, Waltham, MA, USA), equipped with a manual injection valve (type 7125 NS Rheodyne, Rohnert Park, CA, USA) and a refractive index detector RID-10A (Shimadzu, Kyoto, Japan). The columns were a PL Aquagel-OH MIXED-H (8 μm, 300 mm × 7.5 mm PL Agilent Technologies, Santa Clara, CA, USA) and an Ultrahydrogel 250 (6 µm, 300 mm × 7.8 mm, Waters, Milford, MA, USA). The mobile phase was MilliQ water, and the separation was performed in isocratic conditions, with a flow rate of 0.7 mL/min; injection volume was 20 µL. The concentrations were determined in relation to a calibration curve prepared with standard solution of mannan from *S. cerevisiae* (0–1000 mg/L).

The insoluble fraction of YDs was determined by weighting. The powders were resuspended in model solution at a concentration of 1% (*w*/*v*), then filtered under-vacuum on pre-weighed 0.45 μm pore size cellulose membranes. The membranes were kept in oven at 60 °C up to constant weight; the difference between final and initial weight was used to calculate the content of total insoluble solids.

### 2.6. Antioxidant Properties of YDs Powders

#### 2.6.1. Radical Scavenging Activity

Radical scavenging activity (RSA) was evaluated by DPPH assay as reported in Da Porto et al. [34], with slight modifications. Briefly, YDs were resuspended in model solution (1 % *w*/*v*); 500 µL of the mixture were transferred into 10 mL Pyrex tubes and added to 2.5 mL of DPPH solution (80 µM in absolute ethanol), vigorously mixed and left in darkness for 60 min; this fraction was used to evaluate the RSA of the whole derivative. The remaining suspension was centrifuged (3000 rpm, 10 min) and 500 µL of the supernatant was subjected to DPPH assay as previously reported, for evaluating the RSA of the soluble fraction of YDs. The absorbance was measured at 515 nm against absolute ethanol, before and after the reaction. The difference between the initial and the final values was used to calculate the RSA, in relation to a calibration curve prepared with standard solution of glutathione (0–650 µM/L).

#### 2.6.2. Content of Glutathione, Cysteine and Reducing Proteins Containing Cysteine Residues by RP-HPLC Analysis

The amount of glutathione (GSH), cysteine (CYS) and reducing proteins containing cysteine residues (RPC) was determined on YDs powders after derivatization by RP-HPLC analysis, as reported by Tirelli et al. [35]. The powders were firstly resuspended in 50 mM of citrate buffer pH 5; after centrifugation, the supernatant was used to determine the amount of GSH and CYS, whereas the pellets were used to assess the possible presence of reducing proteins linked to the insoluble fraction of YDs. The LC system and the operating conditions used are those reported in Voce et al. [22]. The concentrations of antioxidant molecules were calculated in relation to a calibration curve prepared with standard solution of GSH and CYS (0–400 µM/L), whereas RPC were estimated using the equation reported in the reference method [35].

### 2.7. Wine Color Evolution, Total Polyphenols Index and Flavanols

The analytical determinations were performed on wine samples after two and six months of aging on YDs. Wine color (abs 420 nm), browning assay (POM-test) and CieLab parameters were evaluated as described by Voce et al. [29].

Total polyphenol index (TPI) was calculated by measuring the absorbance at 280 nm of diluted wine samples and by multiplying the absorbance for the number of dilution (10). The amount of flavanols was determined following the method described by Zironi et al. [36]; briefly, 0.5 mL of wine were added to 2.5 mL of DAC solution, previously prepared by dissolving 0.1 g of DAC in 75 mL of methanol and 25 mL of hydrochloric acid at 37% *v*/*v*; after 5 min, the absorbance was measured against a blank prepared by replacing the sample with ethanol (10% *v*/*v*). The concentrations were calculated in relation to a calibration curve prepared with standard solutions of catechin (0–25 mg/L).

All the spectrophotometric analyses were performed using a UV/Vis spectrophotometer model V-530 (Jasco Inc, Mary’s Court Easton, MD, USA), in 10 mm path length quartz cuvettes (Kartell S.p.A. Labware Division, Noviglio, MI, Italy).

### 2.8. Aroma Profile by Solid-Phase Microextraction (SPME)-GC-MS

The aroma profile of both YDs powders and wine samples after two and six months of aging was evaluated by SPME-GCMS. For both YDs and wine samples, 20 mL glass vials were used. For YDs, two grams of powders were used, whereas in the case of wines, 10 mL of samples were spiked with 50 μL of ethyl heptanoate (0.0984 g/L in ethanol 96% *v*/*v* as internal standard) and added to sodium chloride (3 g). The glass vials were then sealed with PTFE/silicone septa. The SPME-GCMS system and the operating conditions used for the analysis of volatile profile are those reported in Voce et al. [29]. Volatile profile was then analyzed by SPME-GCMS, using a GC2030 Nexis gas chromatograph, coupled with a QP2020NX mass spectrometer (Shimadzu, Kyoto, Japan) and equipped with a GC autosampler (HTA, Brescia, Italy). Briefly, the samples were pre-conditioned at 40 °C for 15 min and the microextraction was carried out for 15 min at the same temperature using a 2 cm 50/30 μm divinylbenzene/carboxen/polydimethylsiloxane fiber (Supelco, Bellefonte, PA, USA). A J&W DB-Wax capillary column, 30 m × 0.25 mm, 0.25 μm film thickness (Agilent Technologies Inc., Santa Clara, CA, USA) was used for the GC separation, with the following operating conditions: 40 °C for 1 min, then 4 °C/min, up to 240 °C, with a final holding of 15 min. Injection was performed in splitless mode with 60 s of splitless time; injection port and transfer line were set at 250 °C and 240 °C, respectively. Carrier gas was helium, at a linear flow rate of 35 cm/s. Electron impact mass spectra were recorded at 70 eV and the identification of volatile compounds was tentatively carried out by comparing their mass spectrum with those reported in spectrum library NIST 20. For each detected compound, linear retention index was also calculated based on the retention times of n-alkanes and compared with those reported in the literature.

### 2.9. Statistical Analysis

The results obtained represent the means and the standard deviations of three repeated trials. Homogeneity of variance was evaluated by Brown-Forsythe and Cochran, Hartley, and Bartlett test; ANOVA and Tukey HSD test were carried out for all the parameters analyzed and the differences were considered significant at *p* < 0.05. Concerning aroma compounds, factorial analysis (FL > 0.7) and principal component analysis (PCA) were also performed. Relationships among the parameters were investigated using Pearson correlation coefficient and the statistical significance was fixed at *p* < 0.05. All elaborations were carried out by the software Statistica for Windows Version 8.0 (StatSoft, Tulsa, OK, USA).

## 3. Results

### 3.1. Characterization of YDs Powders

The chemical characterization of yeast derivatives’ powders was carried out in model solution, on both the soluble and insoluble fraction. Means and standard deviations of three repeated trials were calculated and the effect of yeast strains (Y), methods for inducing autolysis (T) and the interaction between the two factors (Y × T) are reported in Table 1. The results are discussed in depth in their respective, following sections.

#### 3.1.1. Amino Acids and Soluble Proteins, Polysaccharides and Insoluble Solids

The release of free amino acids and soluble proteins was affected by both the yeast strains and the treatments performed for inducing autolysis, with the highest values observed for S_US (32 mg/g of amino acids and 215 mg/g of proteins), thus resulting significantly different from all the other samples; however, the mean content of soluble proteins detected in T_US was not negligible (87 mg/g) (Table 1). Considering the content of soluble polysaccharides, even in this case an effect of the yeast strain and treatment was observed, with the best results obtained when *T. delbrueckii* was used as starting microorganism or when ultrasounds (US) and traditional methods (THERM and ENZ) were performed. In fact, the highest polysaccharide content was found in T_ENZ (178 mg/g), followed by S_THERM (117 mg/g) and S_US (116 mg/g), resulting significantly different from all the other samples (Table 1). Lastly, the amount of insoluble solids was affected by both strain and treatment, with derivatives obtained starting from *T. delbrueckii* and treated by thermal inactivation (THERM) or high hydrostatic pressure (HHP) being characterized by the highest mean values (Table 1). Considering all the samples, the highest concentration of the insoluble fraction was found in derivatives obtained by enzyme addition (ENZ) and THERM in the case of *S. cerevisiae* (616 mg/g and 678 mg/g, respectively), and by HHP, THERM and US in the case of *T. delbrueckii* (823 mg/g, 659 mg/g and 619 mg/g, respectively). As expected, S_US and T_ENZ showed the lowest amount of insoluble fraction (289 mg/g and 350 mg/g, respectively) since these products were characterized by the highest mean values of intracellular and soluble molecules (amino acids, proteins and polysaccharides).

#### 3.1.2. Antioxidant Properties

The antioxidant properties of YDs were evaluated in terms of both radicals scavenging activity (RSA, expressed as µmol of glutathione) and content of cysteine, glutathione and reducing proteins containing cysteine residues (RPC) (Table 1).

Starting from RSA, no effect of the yeast strain was observed on neither the whole YDs’ powder nor the soluble fraction. On the other hand, the RSA was significantly affected by the treatment performed, with HHP determining the lowest values, whereas THERM, ENZ and US have given similar results. Considering all the samples, the highest values of RSA evaluated on the whole autolysate were obtained by traditional methods in the case of *T. delbrueckii* (60.6 µmol/g and 51.9 µmol/g in T_ENZ and T_THERM, respectively), whereas US and THERM determined the highest values in the case of *S. cerevisiae* (51.1 µmol/g and 46.8 µmol/g, respectively). Furthermore, the antioxidant activity of YDs seemed to be mainly related to the soluble fraction of the powders, since it represented from 60% to 80% of the total radical scavenging activity.

Concerning antioxidant molecules, cysteine, glutathione and RPC, the content was affected by both yeast strain and treatment. In particular, the use of *T. delbrueckii* as starting microorganism treated by ENZ and US seems to enhance the content of cysteine in the resulting derivatives, whereas the highest concentrations of glutathione and RPC were detected when derivatives were obtained starting from *S. cerevisiae* treated by HHP and ENZ. Generally, the highest concentrations of cysteine were detected in T_ENZ (3.1 µmol/g), S_US (2.9 µmol/g) and T_US (2.5 µmol/g), that resulted significantly different from all the other samples. Glutathione was found in higher amounts in S_HHP (9 µmol/g) and T_ENZ (7.2 µmol/g), whereas derivatives obtained starting from S. cerevisiae (s) treated by enzyme addition or high hydrostatic pressure (S_ENZ and S_HHP, respectively), resulted the most characterized in terms of RPC content linked to the insoluble fraction, with amounts of 66.5 µmol/g and 49.8 µmol/g, respectively. Concerning *T. delbrueckii*, the content of RPC in derivatives obtained from this strain was preserved only in those subjected to HHP treatment (35.7 µmol/g).

#### 3.1.3. Aroma Profile

**Figure 1:**
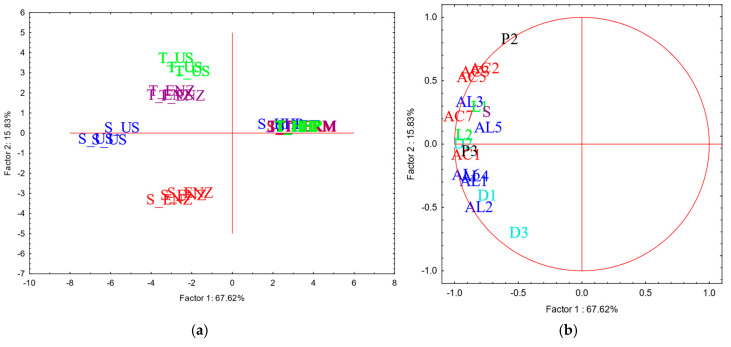
Results of PCA carried out on absolute area of volatile compounds detected in headspace of yeast derivatives powders. Projection of case (samples) (**a**) and variables (volatile compounds) (**b**) on factor-plan were reported. Factor Loadings (FLs) were calculated by factorial analysis, and the most relevant variables were selected for marked FL > 0.7. S: *S. cerevisiae*; T: *T. delbrueckii*; ENZ: enzyme addition; THERM: thermal inactivation; HHP: high hydrostatic pressure: US: ultrasound. AC: acids (red); AL: alcohols (blue); D: diols (light blue); L: lactones (light green); P: pyrazines (black); S: sulfur compounds (violet). Numbers reported after letters indicate specific aroma compound (for details, see Table S2).

A total of twenty-eight volatile compounds were tentatively identified and listed in Table S1 (Supplementary Materials), whereas the results of the PCA carried out on the absolute area of the volatile compounds detected is reported in Figure 1a,b.

The volatile profile of the YDs seemed to be strongly affected by the treatments performed for inducing autolysis. In general, derivatives obtained by ultrasounds (US) and enzyme addition (ENZ) were mostly characterized in terms of aroma compounds, for both the yeast strains, whereas thermal inactivation (THERM) and high hydrostatic pressure (HHP) allowed to obtain products with potential low odor impact. While the concentration seemed to be dependent on the treatment, the type of aroma compounds seemed to be influenced by the strain: acids mainly characterized derivatives obtained from T, whereas alcohols, diols and lactones were mostly present in derivatives from S.

### 3.2. Effect of YDs Addition on Wine Evolution and Volatile Profile During Aging

#### 3.2.1. Color Evolution, Total Polyphenols Index and Flavanols

The effect of the addition of YDs on wine color evolution, predisposition to browning (POM-test), total polyphenols index (TPI) and flavanols after two and six months of aging is reported in Table 2.

Considering the data referred to wines after two months of aging, wines with S_ENZ and T_US showed a behavior similar to control wines for all the parameters evaluated. In general, almost all the samples with YDs showed a good color evolution, with values of absorbance at 420 nm ranging from 0.06 to 0.07, quite similar to control wines (0.05); only wines with S_HHP (0.08), T_ENZ (0.08) and T_HHP (0.09) showed the highest values of wine color, thus resulting significantly different from the control samples. This trend is also confirmed by the results of CieLab analysis, with wine samples with S_HHP, T_ENZ and T_HHP reporting the lowest values of lightness (L* of 97.59, 97.58 and 96.51, respectively) and the highest values in terms of red (+a*) and yellow (+b*) hue. Concerning the predisposition to browning, evaluated by POM-test, this index is related to the potential oxidizability of wine, with lower values corresponding to lower oxidizability. The highest mean value of POM-test was observed in control wines, even if no significant differences were found in comparison to almost all the samples. Even in this case, only wines with T_HHP resulted different from control wine, with a POM-test value of 36%. No significant differences were observed among wine samples concerning TPI, even if the lowest mean value was found for T_HHP (5.1). Lastly, regarding to the content of flavanols, no significant differences were observed among almost all the wines, with values ranging from 6.8 mg/L (S_US) to 8.1 mg/L (S_THERM); only wine with T_ENZ (6.4 mg/L) resulted significantly different from T_THERM (8.3 mg/L).

After six months of aging (Table 2), the only statical differences among the samples was observed for the b* parameter, corresponding to the yellow hue. In general, a slight color loss was observed in almost all wines aged on YDs, except for S_US, T_ENZ and T_HHP (0.07, comparable to CON). This trend was confirmed by CieLab results; no differences were observed concerning lightness (L*) and red-blue hue (a*). The lowest values in yellow color (b*) were detected in S_ENZ (3.85), S_THERM (3.97) and T_HHP (4.08), whereas S_US showed the highest intensity (4.88); for the latter parameter, all the above-mentioned samples resulted statistically different from control wine (4.53). No differences were observed for POM-test, TPI and flavanols, compared to control samples. However, S_ENZ and T_US showed the highest values in terms of oxidizability potential (POM-test values of 98% and 92%, respectively); in both the samples, the higher color decrease was also coupled with lower mean values of TPI (4.9 and 4.6 in S_ENZ and T_US, respectively) and of flavanols, only in the of S_ENZ (7.1 mg/L).

#### 3.2.2. Release of Polysaccharides

**Figure 2:**
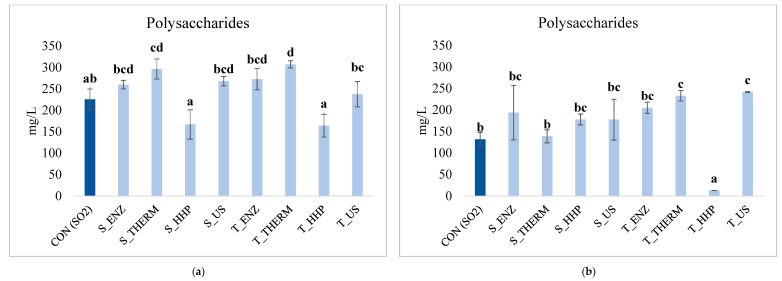
Polysaccharides (mg/L) detected in wines after two (**a**) and six (**b**) months of aging. Data are means and standard deviations of three repeated trials. Different letters mark significant differences among samples, according to ANOVA and Tukey HSD test (*p* < 0.05). CON (SO_2_): control; S: *S. cerevisiae*; T: *T. delbrueckii*; ENZ: enzyme addition; THERM: thermal inactivation; HHP: high hydrostatic pressure; US: ultrasounds.

The effect of YDs addition on the amount of polysaccharides released into the wines during aging is reported in Figure 2 (a, after two months; b, after six months).

The addition of YDs to the wines caused an increase in the content of total polysaccharides during aging, particularly evident after two months of aging (Figure 2a). Wines with YDs obtained from both the yeast strains treated by thermal inactivation showed the highest amount of polysaccharides (297 mg/L and 307 mg/L in S_THERM and T_THERM, respectively), thus resulting significantly different from the control (225 mg/L). Conversely, wines with YDs from both the strains treated by HHP showed the lowest concentration in polysaccharides, with an amount of 166 mg/L and 164 mg/L detected in *S. cerevisiae* (S_HHP) and *T. delbrueckii* (T_HHP), respectively. For the remaining samples, the concentration of polysaccharides was quite similar, ranging from 237 mg/L (T_US) to 273 mg/L (T_ENZ).

After six months of aging (Figure 2b), a general decrease in the content of polysaccharides was observed in almost all the samples, except for wines with S_HHP and T_US which showed a slight increase, reaching a final concentration of 178 mg/L and 242 mg/L, respectively. The above-mentioned samples resulted significantly different from control wines (132 mg/L), and wines with T_HHP also differed from all the other samples due to the lowest content of polysaccharides detected (13 mg/L).

#### 3.2.3. Aroma Profile

**Figure 3:**
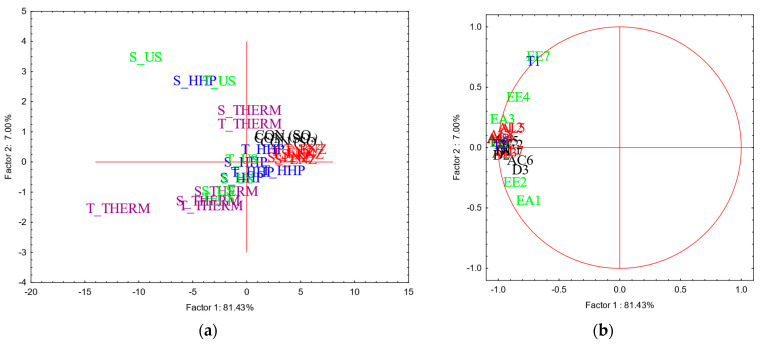
Results of PCA carried out on the concentration (µg/L) of volatile compounds detected in the headspace of wines after two months of aging. Projection of case (samples) (**a**) and variables (volatile compounds) (**b**) on factor-plan are reported. Factor Loadings (FLs) were calculated by factorial analysis, and the most relevant variables were selected for marked FL > 0.7. CON (SO2): control; S: *S. cerevisiae*; T: *T. delbrueckii*; ENZ: enzyme addition; THERM: thermal inactivation; HHP: high hydrostatic pressure: US: ultrasound. AC: acids (black); AL: alcohols (red); D: diols (black); EE: ethyl esters (light green); EA: acetate esters (light green); AE: other esters (light green); EI: aging esters (dark green); S: sulfur compounds (violet); T: terpenes (blue). Numbers reported after letters indicate the specific aroma compound (for details, see Table S3).

**Figure 4:**
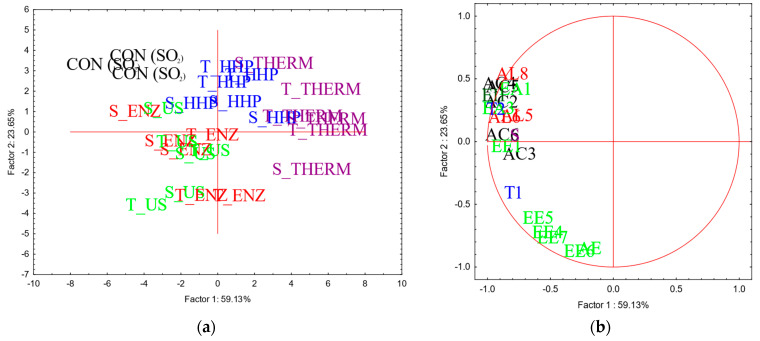
Results of PCA carried out on concentration (µg/L) of volatile compounds detected in headspace of wines after six months of aging. Projection of case (samples) (**a**) and variables (volatile compounds) (**b**) on factor-plan are reported. Factor Loadings (FLs) were calculated by factorial analysis, and the most relevant variables were selected for marked FL > 0.7. CON (SO2): control; S: *S. cerevisiae*; T: *T. delbrueckii*; ENZ: enzyme addition; THERM: thermal inactivation; HHP: high hydrostatic pressure: US: ultrasound. AC: acids (black); AL: alcohols (red); EE: ethyl esters (light green); EA: acetate esters (light green); AE: other esters (light green); EI: aging esters (dark green); S: sulfur compounds (violet); T: terpenes (blue). Numbers reported after letters indicate the specific aroma compound (for details, see Table S4).

A total of thirty-eight volatile compounds were tentatively identified in the headspace of aged wines (Table S1, Supplementary Materials). The results of PCA carried out on the concentrations (µg/L) of aroma compounds detected into the wines after two and six months of aging are reported in Figure 3 and Figure 4, respectively, whereas means and standard deviations, and the results of statistical analysis, are reported in Supplementary Materials (Tables S3 and S4).

After two months of aging (Figure 3a,b), the aroma profile of aged wines was not defined, with different behavior even among replicates, probably because the time considered was not enough for the aroma profile to reach a stable state.

At the end of aging (six month), the volatile profile of the wines was quite different compared to the previous sampling time, with a well-defined aroma profile; the greater differentiation among the samples has allowed to better clarify the impact of the different YDs on the volatile profile of the resulting wines (Figure 4a,b).

In general, the volatile profile of wines seems to be mostly affected by the treatments performed to obtain YDs, rather than yeast strains; wines with YDs obtained from both the strains subjected to ultrasounds treatment (US) and enzyme addition (ENZ) showed the most characterized volatile profile, similar to control wines, whereas the addition of YDs obtained from thermal inactivation (THERM) determined the poorest one. Lastly, YDs previously obtained by high pressure (HHP) partially impacted on wine aroma, with a profile intermediate between the other two groups. Concerning the type of aroma compounds, higher mean concentrations of volatile fatty acids and alcohols were found in control wines, whereas esters mainly characterized wines with S_ENZ, T_ENZ, S_US and T_US. No correlation between aroma compounds of YDs powders and the respective wines were found, whereas a negative correlation between insoluble solids and 3-methylbutyl octanoate was detected (r = −0.78). Furthermore, a positive correlation was found between polysaccharides and some ethyl esters: ethyl octanoate (r = 0.77), ethyl nonanoate (r = 0.80), ethyl decanoate (r = 0.89), 3-methylbutyl octanoate (r = 0.90) and ethyl-9-decenoate (r = 0.81).

## 4. Discussion

### 4.1. Effect of Strain and Technology on the Chemical Composition of YDs 

#### 4.1.1. Content of Free Amino Acids and Soluble Proteins, Polysaccharides and Insoluble Solids

Considering the concentration of soluble molecules in the YDs powders, i.e., amino acids, proteins and polysaccharides, the higher cell permeability of cell walls and membranes induced by enzyme addition [37] or by ultrasounds [38,39] may enhance the release of intracellular components, thanks to the induction of autolysis and degradation phenomena, consequentially increasing the concentration of soluble molecules. Furthermore, the use of US for processing fermentation lees has enhanced the release of proteins and soluble colloids during wine aging on lees, with results comparable to enzyme addition [28]. This might explain the highest mean values of nitrogen compounds (amino acids and proteins) detected in S_US and T_US, and of polysaccharides in S_US and T_ENZ. Furthermore, the concentration of proteins detected in S_ENZ (51 mg/g) and T_ENZ (71 mg/g) was in line with those of commercial yeast extracts also obtained by enzyme-assisted lysis [40].

Autolysates obtained from *S. cerevisiae* after thermal inactivation have showed concentration of soluble polysaccharides higher than those obtained from the same strain subjected to enzyme addition or mechanical disruption [11]; this scientific evidence may confirm the trend here observed, with higher content of polysaccharides detected in S_THERM (117 mg/g), compared to S_ENZ (17 mg/g) and S_HHP (11 mg/g). As expected, derivatives characterized by the highest concentration of soluble compounds, namely S_US and T_ENZ, as previously discussed, showed the lowest amount of insoluble fraction, probably due to a more intense cell degradation that occurred in these samples. On the other hand, YDs obtained by thermal inactivation (THERM) and high hydrostatic pressure (HHP) tendentially showed the highest content of insoluble fraction. The higher temperature reached during thermal treatments generally used for the extraction of mannoproteins [41], as well as pressure above 600 MPa during high-pressure treatments may cause the inactivation of intracellular enzyme, consequentially reducing or preventing the autolytic process [42]. This might explain the tendential higher concentration of insoluble solids detected in derivatives obtained by THERM and HHP.

#### 4.1.2. The Antioxidant Properties of YD Powders

Regarding the antioxidant properties of YDs, the radical scavenging activity was mostly affected by the treatment performed for inducing lysis, whereas the concentrations of glutathione, cysteine and RPC were affected by both the yeast strain and treatment.

The effect of the strain used on the antioxidant properties of the respective derivatives might be due to a different sulfur metabolism linked to glutathione production [43] or to a different behavior in the synthesis of proteins linked to the cell walls [44], thus possibly explaining the different values in terms of RSA and content of antioxidant molecules between S and T, as previously discussed. Considering the effect of the treatment on the antioxidant properties of derivatives, Tirelli et colleagues [35] observed that the occurrence of Maillard’s reaction during the manufacturing process of YDs might lead to a lack of sulfur compounds, and this might explain the tendential lower concentration of antioxidant molecules (cysteine, glutathione and RPC) here detected in derivatives obtained by thermal inactivation (e.g., 5 µmol/g in S_THERM and 3.5 µmol/g in T_THERM of glutathione, Table 1). On the other hand, the low processing temperature during HHP treatment as here performed (30 °C) seems to have preserved the content of antioxidant molecules, with derivatives obtained from both the strains treated by this technology the most characterized in terms of glutathione (e.g., 9 µmol/g in S_HHP) together with a good content of RPC (48.8 µmol/g S_HHP and 35.7 µmol/g in T_HHP). Lastly, the potential activity of exogenous enzyme (ENZ) or the possible enhanced proteolytic activity induced by US, as discussed above concerning the content of amino acids and proteins, might have led to a higher protein hydrolysis. This might consequentially lead to a faster release of amino acids and small peptides that seem to exhibit antioxidant properties [45,46]; this might thus explain, at least in part, the values here observed for S_US and T_ENZ in terms of RSA (51.1 µmol/g and 60.9 µmol/g, respectively).

#### 4.1.3. Aroma Profile of YD Powders

Concerning the effect of the strain on the nature of volatile compounds detected in YDs powders, a potential different metabolic behavior might have influenced the different concentration of fatty acids and alcohols in derivatives obtained from T and S, respectively. Since these two groups of aroma compounds derive from sugar and amino acids metabolism [47] and they are then released during autolysis [48], yeast derivatives generally contain such aroma compounds [40].

Concerning the effect of the treatment on the volatile profile of derivatives’ powders, the results here obtained are in line with those previously reported in the literature. Enzyme addition leads to a more characterized volatile profile in the resulting autolysates, with higher concentration of fatty acids [11]; conversely, high-pressure treatments seem to be suitable to obtain products with poor volatile profile, with lower content of fatty acids and alcohols, similarly to thermal treatments or commercial products [49].

Lastly, the concentration of pyrazines was mostly influenced by the treatment, since their formation occurs during manufacturing process due to the Maillard’s reaction [50]. The presence of pyrazines in YDs’ powders have been already reported [40,51], with products obtained by enzyme addition the most characterized [11]. This is in line with the results here obtained, with derivatives obtained by enzyme addition and ultrasounds the most characterized in terms of pyrazine content. However, the tendential higher concentration of such compounds in the above-mentioned products might be related to the fact that such products were also characterized by the higher mean content of nitrogen compounds, thus possibly determining the occurrence of Maillard reaction, at least at the early stages.

### 4.2. Effect of the Addition of YDs on Wine Chemical Composition and Volatile Profile

#### 4.2.1. Wine Color Evolution, Total Polyphenols Index and Flavanols Content

After two months of aging, the addition of yeast derivatives did not significantly affect the color evolution, total polyphenols index (TPI) and flavanols compared to control. However, the tendential lower value of TPI (5.1) together with the higher value in terms of color (0.09) and the lower oxidizability potential (36%) observed in T_HHP might be due to oxidation phenomena. Conversely, in the case of T_ENZ, the lower concentration of flavanols, together with a good oxidizability potential observed in such samples (55%) might be linked to absorption phenomena of these phenolic fraction by polysaccharides or the insoluble fraction of YDs, thus protecting them from oxidation phenomena.

In previous experiments [49], an increase in wine color and a decrease in oxidizability potential were observed in wines with YDs after one month from their addition, with sulfur dioxide the most effective in protecting wine from oxidation. This evidence may confirm the results here observed, especially as concern wines with T_HHP, S_HHP and T_ENZ. Furthermore, the addition of YDs determined an increase in wine color in the first months after their addition, followed by a decrease [5], without a significant reduction in total polyphenols content after six months of aging [8], confirming the trend here observed after six months. In general, it would be said that the best results were obtained in wines with S_ENZ, at both sampling times. In addition to mannoproteins [5] and glutathione [46,52], other compounds may exhibit antioxidant properties, such as small peptides [53] or compounds linked to cell wall fraction [35,54]. Considering the chemical composition of YDs (Table 1), S_ENZ showed a mean content of glutathione of about 4 μmol/g, lower than those detected in S_HHP (9 μmol/g) and in T_ENZ (7.2 μmol/g). The occurrence of a gradual, slow release of glutathione or other antioxidant molecules during wine aging together with the highest amount of reducing proteins detected in S_ENZ (66.5 μmol/g) might explain, at least in part, the better behavior of this YD towards wine color evolution and protection against oxidation. Furthermore, it has been already reported that the insoluble fraction of lees [55] and of yeast derivatives [2,8] might adsorb the phenolic fraction, thus preserving them from oxidation. The non-negligible content of insoluble fraction that characterized S_ENZ (616 mg/g, see Table 1) might explain the lowest mean values in total polyphenols index and flavanols detected in the resulting aged wines, further confirming the better behavior and the good protection against wine oxidation of such derivative.

#### 4.2.2. Content of Polysaccharides After Aging on YDs

Considering the chemical composition of YDs powders (Table 1), it could be said that in general YDs richer in polysaccharides have determined a tendential higher release of such compounds in the respective added wines, despite of the decrease at the end of aging period. Similarly, the lowest concentration found in wines with derivatives obtained from both the strains treated by high hydrostatic pressure might be explained by the lowest content in polysaccharidic fraction in the respective derivatives (11 mg/g in S_HHP and 21 mg/g in T_HHP, Table 1). Lastly, even if S_ENZ was characterized by a low content of polysaccharides (17 mg/g, Table 1), the amount detected into the respective added wines after aging was comparable to the other samples. During wine aging on lees, the occurrence of autolysis leads to an enrichment of wine in polysaccharides, also depending on the yeast strains used, with interesting results obtained by non-*Saccharomyces* spp. [19]. This might explain the good release of such compounds in wines with YDs obtained starting from *T. delbrueckii*, underlighting its good aptitude to be used for YDs production, comparable to *S. cerevisiae*. In the present study, the initial increase of polysaccharides was followed by a reduction when the longer period of aging (six months) has been considered. This decrease has been previously observed by other authors [8] and it may be probably due to degradation phenomena [56] or to the formation of greater complexes with other wine colloidal substances that may precipitate [57]. As for YDs manufacturing process, the efficacy of ultrasound in favoring the release of polysaccharides into the wine during AOL has been reported [20], with results comparable to those obtained by enzyme addition [28]. In the present study, the concentration of polysaccharides in wines with YDs obtained by applying US was quite similar to traditional methods, making it a suitable technique for producing YDs. Regarding HHP, even if the YDs powder were not characterized by the greatest concentration in polysaccharides, the amount detected into the wines after the aging period was quite comparable to the other samples, especially as concern wines with S_HHP (178 mg/L). Although pressure above 600 MPa may inactive enzymes, a residual enzymatic activity might occur [42], thus favoring the release of cell wall polysaccharides during aging. Furthermore, the hydrolysis of polysaccharides with high molecular weight [56], together with the fact that the higher concentration of polysaccharides in YDs does not always correspond to the highest release into the wines, might explain the trend here observed for wines with YDs obtained by HHP and THERM. Considering above, HHP may also be applicable for YDs production, as alternative to traditional methods.

#### 4.2.3. Aroma Profile of Wines After Aging on YDs

After two months of aging, the volatile profile of aged wines was not well-defined. In general, it would be said that derivatives obtained starting from both the strains subjected to thermal and ultrasounds treatments tendentially showed the most characterized volatile profile, whereas control wines and wine samples with YDs obtained by enzyme addition (S_ENZ and T_ENZ) together with T_HHP showed the poorest aroma profile. These results are in line with those reported by Comuzzo et colleagues [11]; the authors observed that wines with YDs obtained by thermal inactivation and mechanical disruption showed the most characterized volatile profile and a more intense aroma perception, and even the respective powders were the poorest characterized in terms of volatile compounds.

Considering the volatile profile of wines at the end of the aging period considered, the results here obtained agree with those reported in the literature; the addition of derivatives may enhance floral and fruity notes due to an increased volatility of some esters, when the products were added at a concentration of 200 mg/L (similar to the dosage used in the present study, 300 mg/L) [40]. Furthermore, the presence of polysaccharides, especially mannoproteins, seems to increase the volatility of some aroma compounds (i.e., alcohols and esters), dependent on the type of products and glycosylation degree during a short contact time [9,12]. Conversely, a more prolonged contact time may determine the release of odor-active compounds from YDs powders (i.e., pyrazines) as well as the retention of wine volatile compounds by the insoluble fraction of YDs [51,58]. Some authors also observed that volatile compounds previously retained by YDs may then be released into the wines during more prolonged contact times, up to six months [3,59]. This scientific evidence may confirm the trend here observed; wines with THERM and HHP showed the poorest volatile profile, probably due to the high concentration of insoluble solids detected in the respective YDs powders that may have retained some wine volatile compounds (Table 1). No pyrazines were detected in wines with YDs obtained by US and ENZ even if the respective powders contained such compounds (Figure 1a,b), similarly to those observed by other authors [11]. On the other hand, the higher mean concentration of polysaccharides released into the wine with YDs obtained by ENZ and US might have firstly determined a retention of most of volatile compounds during the short aging period, explaining the poorest volatile profile of the respective wines after two months of aging (Figure 3a,b). A subsequent release of the retained volatile compounds might occur during a more prolonged contact time, thus explaining the more intense volatile profile and the tendential higher concentration of esters detected in the above-mentioned added wines after six months of aging, comparable to control wines.

## 5. Conclusions

The chemical composition of yeast derivatives may be managed by using specific strains and by performing the most suitable treatments for inducing autolysis, consequentially obtaining products with specific features. While the starting composition of YDs did not affect wine evolution, the manufacturing process and consequentially the chemical composition of YDs powders mostly affected the wine volatile profile. An enhancement of fruity and floral notes may be achieved by adding YDs obtained by ultrasounds and enzyme addition, whereas the retention of undesired odor-active compounds, e.g., fatty acids, may be possible by using YDs obtained by thermal inactivation and high hydrostatic pressure. *T. delbrueckii* seems to be a good candidate to produce YDs, comparable to *S. cerevisiae*, whereas emerging technologies may be a good alternative to replace the traditional methods during the manufacturing process. This may allow to tailor the chemical composition of yeast derivatives and to standardize their production process. Further investigations are needed to assess the effect on evolution, volatile and sensory profile of different wines; moreover, the addition of YDs during alcoholic fermentation may also be evaluated with the aim to enrich wines of polysaccharides and antioxidant compounds already during the first steps of winemaking. The use of YDs with specific enological characteristics, such as high content of polysaccharides and antioxidant compounds or low odor impact, might allow to reduce the amount of sulfur dioxide or to modulate the wine volatile profile, with a potential reduction of aging time and consequently of the costs of the overall winemaking process. However, further experiments will be carried out to evaluate these aspects, at industrial and winery scale.

## Figures and Tables

**Table 1:** Effect of yeast strains (Y), treatments (T) and interaction between two factors (Y × T) on chemical composition of yeast derivative powders in model solution. Data are means and standard deviations of three repeated trials and concentrations are expressed per gram of powder. Different letters within the same row mark significant differences among groups, according to two-way ANOVA and Tukey HSD test (*p* < 0.05). S: *S. cerevisiae*; T: *T. delbrueckii*; ENZ: enzyme addition; THERM: thermal inactivation; HHP: high hydrostatic pressure: US: ultrasound. RSA: radical scavenging activity expressed in µmol of glutathione; RPC: reducing proteins containing cysteine residues. nd: not detected.

				*S. cerevisiae*																*T. delbrueckii*															
				S_ENZ				S_THERM				S_HHP				S_US				T_ENZ				T_THERM				**T_HHP**				**T_US**			
Parameters	Y	T	Y × T	Mean	±	SD		Mean	±	SD		Mean	±	SD		Mean	±	SD		Mean	±	SD		Mean	±	**SD**		**Mean**	±	**SD**		**Mean**	±	**SD**	
Free amino acids (mg/g)	***	***	***	10	±	1	a	5	±	0	a	9	±	2	a	32	±	5	b	5	±	4	a	3	±	0	a	5	±	1	a	4	±	0	a
Soluble proteins (mg/g)	***	***	**	58	±	3	a	62	±	4	a	53	±	13	a	215	±	37	b	71	±	66	a	49	±	4	a	33	±	8	a	87	±	17	a
Soluble polysaccharides (mg/g)	***	***	***	17	±	4	a	117	±	10	c	11	±	1	a	116	±	20	c	178	±	9	d	62	±	6	b	21	±	3	a	84	±	1	b
Total insoluble solids (mg/g)	***	***	***	616	±	49	cd	678	±	7	de	492	±	82	bc	289	±	2	a	350	±	9	ab	659	±	40	d	823	±	10	e	619	±	112	cd
RSA total fraction (µmol/g)		**	***	29.4	±	1.9	a	46.8	±	6.7	bcd	36.0	±	4.0	abc	51.1	±	7.4	cd	60.6	±	7.1	d	51.9	±	5.9	cd	31.3	±	3.4	ab	36.1	±	7.5	abc
RSA soluble fraction (µmol/g)		***	***	24.6	±	2.4	a	28.5	±	4.5	ab	28.2	±	6.0	ab	41.6	±	0.8	c	41.1	±	1.9	c	37.6	±	4.6	bc	18.7	±	0.3	a	22.6	±	4.2	a
Cysteine (µmol/g)	**	***	***	1.6	±	0.0	a	1.8	±	0.1	a	1.7	±	0.0	a	2.9	±	0.4	bc	3.1	±	0.2	c	1.7	±	0.1	a	1.6	±	0.0	a	2.5	±	0.2	b
Glutathione (µmol/g)	***	***	***	4.0	±	0.4	ab	5.0	±	0.5	b	9.0	±	0.5	d	3.6	±	0.4	ab	7.2	±	0.8	c	3.5	±	0.3	a	3.3	±	0.3	a	4.2	±	0.7	ab
RPC (µmol/g)	***	***	***	66.5	±	2.3	d	33.9	±	2.0	b	49.8	±	2.4	c	40.5	±	5.5	b	1.3	±	0.5	a	nd			a	35.7	±	3.7	b	nd			a

** *p* < 0.01; *** *p* < 0.001.

**Table 2:** Color evolution (abs 420 nm and CieLab parameters, L*, a*, b*), predisposition to browning (POM-test), TPI and flavanols content of wines after two and six months of aging on YDs. Data are means and standard deviations (SD) of three repeated trials. Different letters within the same column mark significant differences among samples, according to ANOVA and Tukey HSD test (*p* < 0.05). Statistical analysis was carried out separately, in relation to aging time. CON (SO_2_): control; S: *S. cerevisiae*; T: *T. delbrueckii*; ENZ: enzyme addition; THERM: thermal inactivation; HHP: high hydrostatic pressure: US: ultrasound.

Sample	Aging Time (Months)	Color (abs 420 nm)				L*				a*				b*				POM-Test (%)				TPI			Flavanols (mg/L)			
		Mean	±	SD		Mean	±	SD		Mean	±	SD		Mean	±	SD		Mean	±	SD		Mean	±	SD	Mean	±	SD	
CON (SO_2)_	2	0.05	±	0.01	a	99.45	±	0.46	b	−0.60	±	0.02	a	3.88	±	0.06	ab	100	±	39	b	5.7	±	0.1	7.0	±	0.1	ab
S_ENZ		0.06	±	0.01	ab	98.66	±	0.60	b	−0.50	±	0.03	abc	3.73	±	0.09	a	82	±	29	ab	5.6	±	0.1	7.0	±	0.4	ab
S_THERM		0.07	±	0.00	ab	98.18	±	0.28	ab	−0.52	±	0.03	abc	3.75	±	0.04	a	41	±	12	a	5.7	±	0.1	8.1	±	0.3	Ab
S_HHP		0.08	±	0.01	bc	97.59	±	1.15	ab	−0.55	±	0.02	ab	3.85	±	0.08	a	55	±	22	ab	5.9	±	0.3	6.9	±	0.4	Ab
S_US		0.07	±	0.00	abc	98.24	±	0.26	ab	−0.47	±	0.05	bc	4.66	±	0.03	e	57	±	15	ab	5.2	±	0.2	6.8	±	1.2	Ab
T_ENZ		0.08	±	0.00	bc	97.58	±	0.29	ab	−0.42	±	0.05	c	4.48	±	0.19	de	55	±	16	ab	5.5	±	0.3	6.4	±	0.8	A
T_THERM		0.07	±	0.01	ab	98.32	±	0.64	ab	−0.49	±	0.05	abc	4.21	±	0.10	cd	59	±	16	ab	5.8	±	0.1	8.3	±	0.3	B
T_HHP		0.09	±	0.02	c	96.51	±	1.22	a	−0.41	±	0.09	c	4.25	±	0.13	cd	36	±	17	a	5.1	±	1.2	6.8	±	0.4	Ab
T_US		0.06	±	0.01	ab	98.75	±	0.36	b	−0.56	±	0.03	ab	4.17	±	0.12	bc	79	±	12	ab	5.7	±	0.6	7.0	±	0.9	Ab
CON (SO_2)_	6	0.07	±	0.01		98.56	±	0.64		−0.61	±	0.02		4.53	±	0.06	c	91	±	35		5.3	±	0.3	8.1	±	0.5	
S_ENZ		0.06	±	0.00		98.70	±	0.06		−0.62	±	0.04		3.85	±	0.02	a	98	±	6		4.9	±	0.7	7.6	±	0.9	
S_THERM		0.06	±	0.00		98.99	±	0.12		−0.66	±	0.02		3.97	±	0.05	a	76	±	13		5.3	±	0.2	8.3	±	0.4	
S_HHP		0.06	±	0.00		98.70	±	0.16		−0.64	±	0.02		4.08	±	0.11	ab	81	±	8		5.0	±	0.6	8.5	±	0.4	
S_US		0.07	±	0.00		98.09	±	0.18		−0.61	±	0.02		4.88	±	0.10	d	73	±	13		5.8	±	0.2	8.2	±	0.3	
T_ENZ		0.07	±	0.00		98.06	±	0.38		−0.63	±	0.04		4.50	±	0.09	c	88	±	15		5.6	±	0.3	8.0	±	0.3	
T_THERM		0.06	±	0.00		98.90	±	0.09		−0.66	±	0.00		4.19	±	0.10	abc	80	±	7		5.9	±	0.7	8.4	±	0.4	
T_HHP		0.07	±	0.01		98.29	±	0.86		−0.60	±	0.05		4.32	±	0.29	bc	72	±	25		5.5	±	0.2	7.9	±	0.7	
T_US		0.06	±	0.00		98.40	±	0.16		−0.66	±	0.02		4.46	±	0.05	c	92	±	24		4.6	±	1.9	8.1	±	1.4	

## Data Availability

The original contributions presented in the study are included in the article/Supplementary Materials, further inquiries can be directed to the corresponding author.

## Supplementary Materials

The following supporting information can be downloaded at: https://www.mdpi.com/article/10.3390/foods14040652/s1, Table S1. Volatile compounds tentatively identified by SPME-GC-MS in headspace of yeast derivatives powders (YDs), and in wines after two and six months of aging (W); Table S2. Results of semi-quantitative analysis carried out on absolute area of volatile compounds detected in headspace of yeast derivatives’ powders. Table S3. Results of semi-quantitative analysis carried out on concentration (µg/L) of volatile compounds detected in headspace of wines after two months of aging. Table S4. Results of semi-quantitative analysis carried out on concentration (µg/L) of volatile compounds detected in headspace of wines after six months of aging.
