# Supplementary material for: Use of Emerging Technologies and Non-Saccharomyces spp. for Tailoring the Composition of Yeast Derivatives: Effect on White Wine Aging
Source: Foods. 2025 Feb 14;14(4):652. doi: 10.3390/foods14040652 (PMC11854885; doi:10.3390/foods14040652)
Supplement: Supplementary file 1 [file foods-14-00652-s001.zip › foods-3443184-supplementary.pdf]

Table S1. Volatile compounds tentatively identified by SPME-GC-MS in the headspace of the yeast derivatives powders (YD), and in wines after two and six months of aging (W).

Compound	RI[a]	RI (lit)[b]	IM[c]	Sample
ethyl butanoate	1040	1058	MS RI	W
2-methyl-1-propanol	1101	1'114	MS RI	W
3-methyl-1-butanol acetate	1117	1121	MS RI	W
2-methyl-1-propanol	1127	1114	MS RI	YD
3-methyl-1-butanol	1222	1211	MS RI S	YD W
ethyl hexanoate	1243	1230	MS RI	W
hexyl acetate	1298	1305	MS RI	W
2,5-dimethylpyrazine	1327	1316	MS RI	YD
2,6-dimethylpyrazine	1334	1319	MS RI	YD
ethyl lactate	1346	1340	MS RI	YD W
1-hexanol	1362	1359	MS RI S	YD W
cis-3-hexen-1-ol	1390	1386	MS RI	W
2,3,5-trimethylpyrazine	1409	1408	MS RI	YD
ethyl octanoate	1437	1441	MS RI S	YD W
2-ethyl-3,6-dimethylpyrazine	1452	1455	MS RI	YD
acetic acid	1453	1465	MS RI S	YD W
1-heptanol	1466	1461	MS RI	YD
2-ethyl-1-hexanol	1494	1484	MS RI S	W
ethyl nonanoate	1540	1541	MS RI	W
propanoic acid	1545	1540	MS RI S	YD
2,3-butanediol (levo)	1546	1526	MS RI	YD W
linalool	1552	1552	MS RI	W
1-octanol	1565	1565	MS RI	YD W
2-methylpropanoic acid	1573	1581	MS RI S	YD
2,3-butanediol (meso)	1583	1580	MS RI	YD W
1,2-propanediol	1595	1600	MS RI	YD W
γ-valerolactone	1611	1619	MS RI	YD
γ-butyrolactone	1621	1595	MS RI S	YD W
butanoic acid	1635	1628	MS RI S	YD W
ethyl decanoate	1641	1643	MS RI S	W
3-methylbutyl octanoate	1662	1658	MS RI	W
3-methylbutanoic acid	1675	1666	MS RI S	YD W
diethyl succinate	1678	1694	MS RI	W
ethyl-9-decenoate	1693	1694	MS RI	W
α-terpineol	1699	1692	MS RI	W
methionol	1720	1720	MS RI	YD W
pentanoic acid	1748	1744	MS RI	YD
1-decanol	1769	1769	MS RI	W
2-phenylethyl acetate	1814	1822	MS RI	W
hexanoic acid	1849	1857	MS RI S	YD W
ethyl 3-methylbutyl succinate	1903	1901	MS RI	W
2-phenylethanol	1913	1'922	MS RI S	YD W
2-ethylhexanoic acid	1955	1950	MS RI	YD
1-dodecanol	1966	1940	MS RI	W
diethyl malate	2044	2060	MS RI	W
ethyl tetradecanoate	2046	2043	MS RI	W

(continue)

Table A1. *(continue)*

octanoic acid	2063	2070	MS RI S	YD W
decanoic acid	2273	2278	MS RI	YD W
ethyl hydrogen succinate	2372	2368	MS RI	W

[a] RI: calculated retention index
[b] RI (lit): retention index from literature, source NIST Chemistry WebBook (webbook.nist.gov)
[c] IM: identification method; MS: comparison of mass spectra with those reported in NIST 20 mass spectrum library; RI: comparison of retention index with those reported in literature; S: comparison of mass spectra and retention time with those of standard compounds.

Table S2. Results of the semi-quantitative analysis carried out on the absolute area of volatile compounds detected in the headspace of yeast derivatives' powders. Data are means and standard deviations (SD) of three repeated trials. Different letters within the same row mark significant differences among samples, according to ANOVA and Tukey HSD test ($p < 0.05$). S: *S. cerevisiae*; T: *T. delbrueckii*; ENZ: enzyme addition; THERM: thermal inactivation; HHP: high hydrostatic pressure; US: ultrasounds.

		S. cerevisiae								*T. delbrueckii*							
		ENZ		THERM		HHP		US		ENZ		THERM		HHP		US	
Compound		Mean ± SD		Mean ± SD		Mean ± SD		Mean ± SD		Mean ± SD		Mean ± SD		Mean ± SD		Mean ± SD	
acetic acid	AC1	43218 ± 8095	cd	945 ± 104	a	6736 ± 7744	ab	57344 ± 22384	d	29812 ± 1323	bc	570 ± 66	a	3589 ± 638	a	30453 ± 6223	bc
propanoic acid	AC2	395 ± 146	ab	0 ± 0	a	215 ± 228	a	923 ± 480	bc	1078 ± 27	c	0 ± 0	a	90 ± 23	a	1442 ± 65	c
2-methylpropanoic acid	AC3	22729 ± 1877	b	1502 ± 196	a	3393 ± 3545	a	55844 ± 7323	c	67539 ± 1992	d	2303 ± 589	a	2345 ± 412	a	77494 ± 3521	e
butanoic acid	AC4	1086 ± 63	b	114 ± 10	a	363 ± 363	a	2510 ± 253	c	7507 ± 248	d	223 ± 33	a	398 ± 40	ab	8907 ± 466	e
2-methylbutanoic acid	AC5	14327 ± 802	b	612 ± 127	a	1208 ± 1224	a	32218 ± 3720	c	38953 ± 838	d	907 ± 376	a	1480 ± 216	a	40822 ± 2950	d
pentanoic acid	AC6	nd	ab	nd	a	118 ± 112	a	229 ± 245	a	79 ± 24	a	nd	a	nd	a	121 ± 17	a
hexanoic acid	AC7	305 ± 26	bc	nd	a	99 ± 102	a	553 ± 68	c	362 ± 52	b	nd	a	nd	a	439 ± 29	bc
2-ethylhexanoic acid	AC8	302 ± 347	a	nd	a	nd	a	163 ± 24	a	nd	a	nd	a	nd	a	nd	a
octanoic acid	AC9	nd	a	nd	a	178 ± 308	a	89 ± 10	a	nd	a	nd	a	nd	a	nd	a
decanoic acid	AC10	nd	a	nd	a	53 ± 92	b	nd	a	nd	a	nd	a	nd	a	nd	a
2-methyl-1-propanol	AL1	55051 ± 21058	d	724 ± 222	ab	1184 ± 1146	ab	43441 ± 14289	cd	30518 ± 13245	bcd	nd	a	nd	a	22101 ± 8330	abc
3-methyl-1-butanol	AL2	181811 ± 21200	c	3733 ± 256	a	7523 ± 8123	a	188879 ± 26851	c	47489 ± 2711	b	1036 ± 513	a	1465 ± 235	a	26686 ± 1744	ab
1-hexanol	AL3	966 ± 86	b	nd	a	75 ± 65	a	1030 ± 105	b	1053 ± 121	b	nd	a	nd	a	1564 ± 199	c
1-heptanol	AL4	712 ± 188	bc	nd	a	nd	a	1012 ± 385	c	144 ± 23	a	nd	a	nd	a	402 ± 43	ab
1-octanol	AL5	nd	a	nd	a	nd	a	651 ± 199	b	108 ± 4	a	nd	a	nd	a	138 ± 42	a
2-phenylethanol	AL6	5529 ± 757	d	60 ± 15	a	141 ± 116	a	9379 ± 1193	e	3925 ± 284	c	nd	a	330 ± 42	ab	1665 ± 319	b
2,3-butanediol (levo)	D1	22216 ± 466	c	135 ± 46	a	319 ± 394	a	12470 ± 3706	b	15034 ± 993	b	108 ± 40	a	437 ± 19	a	2506 ± 380	a
2,3-butanediol (meso)	D2	1841 ± 62	bc	nd	a	143 ± 248	a	3284 ± 967	d	2275 ± 147	cd	nd	a	170 ± 143	a	1212 ± 127	b
1,2-propanediol	D3	12223 ± 491	d	96 ± 8	a	619 ± 693	ab	3582 ± 191	c	1469 ± 18	b	nd	a	237 ± 27	a	430 ± 91	a
ethyl lactate	EE1	nd	a	nd	a	nd	a	774 ± 247	ab	1525 ± 806	b	nd	a	nd	a	249 ± 83	a
ethyl octanoate	EE2	152 ± 59	b	nd	a	nd	a	129 ± 74	b	62 ± 2	ab	nd	a	nd	a	nd	a

(continue)

Table A2. *(continue)*

γ-valerolactone	L1		nd		a		nd		a		nd		a	1199	±	497	b	517	±	59	a		nd		a		nd		a	441	±	144	a
γ-butyrolactone	L2	1015	±	29	ab	51	±	11	a	64	±	66	a	2470	±	1111	c	1326	±	172	bc		nd		a	76	±	21	a	1073	±	217	ab
2,5-dimethylpyrazine	P1		nd		a		nd		a		nd		a	212	±	78	b	577	±	32	c		nd		a		nd		a	225	±	68	b
2,6-dimethylpyrazine	P2		nd		a		nd		a		nd		a	397	±	86	b	1016	±	116	c		nd		a		nd		a	1417	±	231	d
trimethylpyrazine	P3	202	±	68	b		nd		a		nd		a	154	±	18	b	163	±	19	b		nd		a		nd		a	138	±	23	b
2-ethyl-3,6-dimethylpyrazine	P4	479	±	318	b		nd		a		nd		a	108	±	95	a	995	±	114	c		nd		a		nd		a	217	±	116	ab
methionol	S		nd		a		nd		a		nd		a	455	±	153	c	290	±	9	b		nd		a		nd		a	67	±	16	a

Table S3. Results of the semi-quantitative analysis carried out on the concentration (µg/L) of volatile compounds detected in the headspace of wines after two months of aging. Data are means and standard deviations (SD) of three repeated trials. Different letters within the same row mark significant differences among samples, according to ANOVA and Tukey HSD test ($p < 0.05$). CON (SO_2): control; S: *S. cerevisiae*; T: *T. delbrueckii*; ENZ: enzyme addition; THERM: thermal inactivation; HHP: high hydrostatic pressure; US: ultrasounds.

		CON (SO_2)	S_ENZ	S_THERM	S_HHP	S_US	T_ENZ	T_THERM	T_HHP	T_US
Compound		Mean ± SD	Mean ± SD	Mean ± SD	Mean ± SD	Mean ± SD	Mean ± SD	Mean ± SD	Mean ± SD	Mean ± SD
acetic acid	AC1	3.9 ± 0.2 ab	4.2 ± 0.8 abc	8.9 ± 2.9 abc	11.9 ± 4.5 c	11.0 ± 1.8 abc	3.5 ± 0.5 a	11.4 ± 5.6 bc	8.0 ± 0.7 abc	8.7 ± 1.9 abc
butanoic acid	AC2	1.0 ± 0.2	0.9 ± 0.1	1.6 ± 0.3	1.6 ± 0.8	1.8 ± 0.7	0.6 ± 0.1	2.4 ± 1.5	0.7 ± 0.6	1.5 ± 0.3
3-methylbutanoic acid	AC3	0.9 ± 0.0	0.9 ± 0.1	2.1 ± 0.5	2.1 ± 0.9	2.9 ± 1.4	0.9 ± 0.1	3.4 ± 1.9	1.1 ± 1.0	15.3 ± 22.3
hexanoic acid	AC4	32.8 ± 1.5 a	26.5 ± 6.8 a	72.2 ± 17.1 ab	69.0 ± 14.5 ab	97.2 ± 36.3 b	27.2 ± 6.1 a	96.4 ± 39.2 b	51.1 ± 8.1 ab	76.9 ± 10.8 ab
octanoic acid	AC5	90.5 ± 6.8 a	72.7 ± 17.7 a	187.2 ± 53.4 ab	176.9 ± 9.5 ab	257.6 ± 92.6 b	75.4 ± 26.9 a	239.0 ± 68.4 b	133.1 ± 34.5 ab	198.3 ± 44.6 ab
decanoic acid	AC6	6.2 ± 0.3 a	4.5 ± 1.2 a	16.0 ± 5.2 ab	13.3 ± 2.8 ab	22.5 ± 6.9 b	4.8 ± 2.2 a	21.5 ± 6.6 b	9.5 ± 5.6 ab	16.8 ± 6.1 ab
2-methyl-1-propanol	AL1	11.1 ± 7.9 b	nd	nd	nd	nd	nd	nd	nd	nd
3-methyl-1-butanol	AL2	362.6 ± 9.0	345.1 ± 55.9	484.0 ± 70.7	489.9 ± 141.0	667.4 ± 307.1	328.1 ± 12.1	691.4 ± 479.4	396.0 ± 48.0	531.1 ± 126.8
1-hexanol	AL3	15.9 ± 0.9 a	13.6 ± 2.3 a	33.4 ± 6.5 ab	33.5 ± 12.6 ab	39.9 ± 9.4 ab	11.5 ± 2.4 a	52.5 ± 34.0 b	23.8 ± 2.1 ab	33.3 ± 2.1 ab
2-ethyl-1-hexanol	AL4	8.7 ± 1.9 ab	6.3 ± 0.9 a	17.6 ± 0.6 ab	8.7 ± 4.2 ab	23.1 ± 10.0 ab	6.2 ± 0.5 a	24.3 ± 13.9 b	6.1 ± 0.8 a	19.6 ± 4.8 ab
1-octanol	AL5	1.2 ± 0.1 a	1.1 ± 0.3 a	3.0 ± 0.3 ab	2.9 ± 0.8 ab	3.5 ± 1.2 ab	1.0 ± 0.3 a	3.9 ± 2.1 b	1.9 ± 0.3 ab	3.0 ± 0.2 ab
1-decanol	AL6	0.6 ± 0.1	0.4 ± 0.1	1.6 ± 0.6	1.4 ± 0.4	1.5 ± 0.6	0.5 ± 0.1	1.6 ± 0.7	0.7 ± 0.6	1.3 ± 0.4
2-phenylethanol	AL7	33.0 ± 1.5 a	29.5 ± 4.8 a	68.2 ± 17.7 ab	67.0 ± 9.8 ab	93.9 ± 38.9 b	27.5 ± 6.2 a	91.9 ± 36.3 b	49.7 ± 8.1 ab	74.7 ± 17.0 ab
2,3-butanediol (levo)	D1	5.5 ± 0.1 ab	5.7 ± 1.3 abc	13.0 ± 5.4 ab	16.2 ± 4.4 b	16.3 ± 4.0 b	4.8 ± 0.6 a	16.7 ± 7.6 b	9.5 ± 2.9 ab	13.1 ± 2.6 ab
2,3-butanediol (meso)	D2	1.6 ± 0.0 ab	1.6 ± 0.2 ab	3.7 ± 1.2 abc	4.0 ± 0.8 abc	4.6 ± 1.2 bc	1.4 ± 0.1 a	5.1 ± 2.4 c	2.3 ± 0.9 abc	3.8 ± 0.5 abc
1,2-propanediol	D3	0.4 ± 0.0	0.4 ± 0.1	0.6 ± 0.2	0.8 ± 0.1	0.7 ± 0.2	0.3 ± 0.1	0.8 ± 0.4	0.3 ± 0.3	0.5 ± 0.2
ethyl hexanoate	EE1	211.9 ± 27.8 a	235.5 ± 31.7 a	481.8 ± 104.9 bcd	341.4 ± 48.2 abc	508.4 ± 21.6 cd	250.7 ± 19.8 ab	588.6 ± 200.0 d	407.1 ± 37.4 abcd	474.7 ± 49.9 bcd
ethyl lactate	EE2	64.5 ± 3.7	66.8 ± 4.8	49.7 ± 5.1	51.8 ± 18.8	58.0 ± 13.9	56.1 ± 7.0	65.1 ± 31.2	35.0 ± 16.7	60.0 ± 16.5
ethyl octanoate	EE3	452.9 ± 62.0 abc	396.2 ± 69.2 a	613.9 ± 35.7 abc	640.5 ± 121.0 bc	662.2 ± 145.7 c	410.3 ± 16.4 ab	605.9 ± 45.3 abc	462.3 ± 99.9 abc	614.9 ± 80.0 abc
ethyl nonanoate	EE4	1.3 ± 0.5	1.6 ± 0.7	2.4 ± 1.1	2.2 ± 1.1	2.3 ± 0.7	1.1 ± 0.5	1.3 ± 1.1	0.8 ± 0.7	2.4 ± 0.6
ethyl decanoate	EE5	43.3 ± 6.3	32.6 ± 1.9	48.1 ± 17.3	57.2 ± 26.0	73.2 ± 54.6	30.7 ± 4.2	57.4 ± 14.4	36.9 ± 14.7	61.0 ± 31.2
ethyl-9-decenoate	EE6	7.6 ± 0.9	5.1 ± 0.4	10.1 ± 3.3	12.2 ± 7.2	12.7 ± 7.8	5.7 ± 0.7	10.8 ± 2.4	7.6 ± 3.2	11.1 ± 4.4
3-methyl-1-butanol acetate	EA1	76.4 ± 5.3	71.5 ± 23.4	139.1 ± 38.0	121.7 ± 15.4	128.1 ± 8.3	69.4 ± 4.2	152.2 ± 78.8	119.6 ± 19.7	103.9 ± 24.6
hexyl acetate	EA2	14.2 ± 2.6	14.5 ± 2.2	24.2 ± 10.9	171.4 ± 255.2	23.7 ± 9.3	15.0 ± 2.2	32.0 ± 8.3	23.4 ± 3.3	30.7 ± 3.0
2-phenylethyl acetate	EA3	3.5 ± 0.2 ab	2.9 ± 0.6 a	7.0 ± 0.6 ab	6.9 ± 1.5 ab	9.5 ± 5.0 b	2.9 ± 0.7 ab	9.5 ± 4.2 ab	4.9 ± 0.2 ab	7.4 ± 1.5 ab
diethyl succinate	EI1	14.2 ± 0.7 a	12.5 ± 2.6 a	34.8 ± 9.1 ab	34.7 ± 6.4 ab	46.4 ± 16.5 b	12.4 ± 2.7 a	47.1 ± 20.6 b	25.9 ± 3.7 ab	37.5 ± 6.1 ab
ethyl 3-methylbutyl succinate	EI2	0.2 ± 0.0 a	0.2 ± 0.1 a	0.6 ± 0.0 a	0.7 ± 0.1 a	0.7 ± 0.4 a	27.9 ± 6.5 b	0.8 ± 0.2 a	0.3 ± 0.3 a	0.7 ± 0.1
diethyl malate	EI3	0.5 ± 0.1	0.6 ± 0.2	1.3 ± 0.8	1.5 ± 0.7	1.6 ± 0.3	0.3 ± 0.2	1.0 ± 0.6	0.6 ± 0.7	1.2 ± 0.5
ethyl hydrogen succinate	EI4	0.5 ± 0.1	0.5 ± 0.1	4.4 ± 4.1	4.0 ± 3.2	5.3 ± 1.4	0.2 ± 0.2	3.5 ± 4.3	1.9 ± 3.1	4.2 ± 3.5

(continue)

Table A3. *(continue)*

3-methylbutyl octanoate	AE	1.2 ± 0.2	1.0 ± 0.2	1.9 ± 1.4	1.4 ± 0.7	3.8 ± 3.9	1.0 ± 0.2	2.8 ± 1.1	1.2 ± 0.1	3.0 ± 2.4
methionol	S	0.3 ± 0.1	0.2 ± 0.1	0.6 ± 0.1	0.5 ± 0.1	0.7 ± 0.3	0.2 ± 0.1	0.8 ± 0.5	0.3 ± 0.2	0.6 ± 0.0
linalool	T1	1.2 ± 0.0	1.0 ± 0.2	1.5 ± 0.4	2.2 ± 1.0	2.3 ± 1.8	1.0 ± 0.2	1.8 ± 0.8	1.5 ± 0.1	1.7 ± 1.0
α-terpineol	T2	0.8 ± 0.0 ab	0.6 ± 0.1 a	2.0 ± 0.4 abc	1.9 ± 0.6 abc	2.6 ± 0.9 bc	0.7 ± 0.1 a	2.9 ± 1.5 c	1.3 ± 0.2 abc	2.2 ± 0.2 abc

AC: acids; AL: alcohols; D: diols; EE: ethyl esters; EA: acetate esters; EI: aging esters; AE: other esters; S: sulfur compounds; T: terpenes.

Table S4. Results of the semi-quantitative analysis carried out on the concentration (µg/L) of volatile compounds detected in the headspace of wines after six months of aging. Data are means and standard deviations (SD) of three repeated trials. Different letters within the same row mark significant differences among samples, according to ANOVA and Tukey HSD test ($p < 0.05$). CON (SO_2): control; S: *S. cerevisiae*; T: *T. delbrueckii*; ENZ: enzyme addition; THERM: thermal inactivation; HHP: high hydrostatic pressure; US: ultrasounds.

		CON (SO_2)	S_ENZ	S_THERM	S_HHP	S_US	T_ENZ	T_THERM	T_HHP	T_US
Compound		Mean ± SD	Mean ± SD	Mean ± SD	Mean ± SD	Mean ± SD	Mean ± SD	Mean ± SD	Mean ± SD	Mean ± SD
acetic acid	AC1	9.9 ± 1.0	9.8 ± 0.9	9.6 ± 0.5	9.3 ± 0.6	9.5 ± 0.9	9.5 ± 0.5	11.6 ± 1.6	10.1 ± 0.8	10.2 ± 0.8
butanoic acid	AC2	1.4 ± 0.1 c	1.2 ± 0.0 bc	0.9 ± 0.1 a	1.1 ± 0.1 ab	1.2 ± 0.0 bc	1.1 ± 0.1 ab	1.0 ± 0.1 ab	1.1 ± 0.1 ab	1.1 ± 0.1 ab
3-methylbutanoic acid	AC3	1.3 ± 0.1	1.3 ± 0.1	1.0 ± 0.0	1.2 ± 0.1	1.2 ± 0.1	1.2 ± 0.1	1.1 ± 0.0	1.1 ± 0.1	1.3 ± 0.3
hexanoic acid	AC4	47.5 ± 1.7 c	39.4 ± 4.0 bc	28.7 ± 5.2 a	35.0 ± 3.6 ab	37.2 ± 3.6 ab	34.2 ± 2.6 ab	28.4 ± 0.4 a	35.8 ± 1.9 ab	37.1 ± 2.0 ab
octanoic acid	AC5	107.0 ± 6.9 c	96.4 ± 9.0 bc	57.1 ± 15.5 a	78.5 ± 11.6 ab	90.0 ± 7.8 bc	75.1 ± 8.5 ab	60.9 ± 3.2 a	93.3 ± 4.0 bc	88.4 ± 7.4 bc
decanoic acid	AC6	9.0 ± 1.0 d	8.5 ± 0.5 cd	3.1 ± 0.6 a	4.8 ± 0.8 ab	7.8 ± 0.2 cd	5.1 ± 0.9 ab	4.2 ± 0.4 ab	6.4 ± 1.2 bc	8.3 ± 1.3 cd
2-methyl-1-propanol	AL1	6.1 ± 1.9	4.4 ± 1.8	6.6 ± 0.7	5.2 ± 2.4	5.7 ± 2.2	5.9 ± 1.7	5.0 ± 1.3	3.4 ± 0.7	5.2 ± 1.5
3-methyl-1-butanol	AL2	367.3 ± 50.6 b	320.4 ± 63.7 b	238.7 ± 139.9 ab	74.1 ± 13.0 a	305.4 ± 49.1 b	311.7 ± 72.4 b	184.0 ± 25.0 ab	210.3 ± 11.7 ab	357.2 ± 52.8 bc
1-hexanol	AL3	37.8 ± 26.5	18.4 ± 1.5	16.8 ± 1.0	18.6 ± 1.6	17.6 ± 1.7	18.7 ± 0.7	16.4 ± 0.5	18.2 ± 0.6	18.5 ± 1.5
cis-3-hexen-1-ol	AL4	0.9 ± 0.1	0.8 ± 0.2	0.8 ± 0.2	1.0 ± 0.1	1.0 ± 0.1	0.9 ± 0.1	0.8 ± 0.1	0.8 ± 0.2	1.0 ± 0.0
2-ethyl-1-hexanol	AL5	33.5 ± 4.6 e	22.9 ± 3.7 d	15.1 ± 1.0 abc	14.4 ± 0.9 ab	22.2 ± 3.2 cd	21.3 ± 0.7 bcd	15.2 ± 2.0 abc	13.9 ± 1.5 a	20.2 ± 1.1 abcd
1-octanol	AL6	1.9 ± 0.1 d	1.7 ± 0.1 cd	1.2 ± 0.2 ab	1.5 ± 0.1 bcd	1.4 ± 0.2 bc	1.5 ± 0.0 bcd	0.9 ± 0.2 a	1.3 ± 0.2 abc	1.5 ± 0.1 bcd
2-phenylethanol	AL7	41.1 ± 3.8 c	40.1 ± 4.5 c	26.2 ± 5.1 a	32.7 ± 5.6 abc	36.0 ± 2.8 abc	28.4 ± 2.9 ab	26.6 ± 2.0 a	38.2 ± 1.3 bc	33.0 ± 1.1 abc
1-dodecanol	AL8	0.8 ± 0.0	0.5 ± 0.0	0.4 ± 0.0	0.8 ± 0.5	0.6 ± 0.1	0.5 ± 0.1	0.6 ± 0.1	0.5 ± 0.1	0.6 ± 0.2
2,3-butanediol (levo)	D1	13.2 ± 1.1 b	11.0 ± 1.2 ab	10.0 ± 1.2 a	11.3 ± 1.4 ab	10.7 ± 0.3 ab	10.4 ± 1.3 ab	10.7 ± 0.6 ab	11.0 ± 0.8 ab	10.7 ± 0.5 ab
2,3-butanediol (meso)	D2	4.5 ± 0.4 b	4.0 ± 0.2 ab	3.2 ± 0.3 a	3.7 ± 0.5 ab	3.9 ± 0.3 ab	3.5 ± 0.2 ab	3.9 ± 0.4 ab	3.7 ± 0.2 ab	4.2 ± 0.9 ab
1,2-propanediol	D3	0.7 ± 0.0	0.6 ± 0.0	0.4 ± 0.0	0.4 ± 0.1	0.6 ± 0.3	0.6 ± 0.1	0.6 ± 0.1	0.6 ± 0.1	0.6 ± 0.2
ethyl butanoate	EE1	10.3 ± 0.9 b	9.5 ± 1.1 ab	7.5 ± 0.7 ab	8.7 ± 1.3 ab	10.0 ± 0.4 b	8.4 ± 0.8 ab	6.9 ± 1.8 a	8.1 ± 1.2 ab	10.3 ± 0.9 ab
ethyl hexanoate	EE2	171.8 ± 66.3 ab	143.4 ± 48.0 ab	199.1 ± 175.1 ab	360.2 ± 26.0 b	111.4 ± 22.5 a	141.4 ± 103.6 ab	257.7 ± 18.0 ab	237.7 ± 71.0 ab	74.5 ± 20.3 a
ethyl lactate	EE3	47.5 ± 4.0 b	37.4 ± 3.8 ab	33.9 ± 1.2 a	34.5 ± 7.0 a	38.1 ± 5.0 ab	36.3 ± 2.6 ab	41.3 ± 3.6 ab	33.9 ± 6.4 a	35.3 ± 3.0 ab
ethyl octanoate	EE4	535.2 ± 69.4 ab	693.4 ± 14.1 c	533.6 ± 103.9 ab	567.8 ± 43.7 abc	667.7 ± 61.9 bc	683.1 ± 28.0 bc	472.4 ± 37.0 a	489.5 ± 24.6 a	718.2 ± 52.6 c
ethyl nonanoate	EE5	2.7 ± 0.0 abc	3.4 ± 0.1 bc	1.2 ± 0.3 ab	0.7 ± 0.2 a	4.1 ± 1.2 c	3.7 ± 0.6 c	1.3 ± 0.1 ab	0.8 ± 0.3 a	4.0 ± 1.8 c
ethyl decanoate	EE6	35.1 ± 3.8 ab	57.4 ± 5.1 abc	40.8 ± 17.3 abc	40.8 ± 1.8 abc	62.3 ± 15.5 abc	63.3 ± 13.0 bc	39.2 ± 9.2 abc	30.5 ± 4.9 a	67.1 ± 16.1 c
ethyl-9-decenoate	EE7	7.0 ± 1.0 abc	10.5 ± 1.4 bc	6.3 ± 2.3 ab	7.3 ± 0.4 abc	10.0 ± 1.7 bc	11.0 ± 2.4 c	5.3 ± 1.2 a	5.3 ± 0.4 a	11.1 ± 1.6 c
ethyl tetradecanoate	EE8	0.7 ± 0.2 ab	0.5 ± 0.1 ab	0.8 ± 0.1 ab	0.7 ± 0.2 ab	0.5 ± 0.0 a	0.5 ± 0.0 a	0.8 ± 0.2 b	0.5 ± 0.0 a	0.7 ± 0.1 ab
3-methyl-1-butanol acetate	EA1	49.1 ± 4.4 b	45.1 ± 4.1 ab	40.5 ± 1.9 ab	45.9 ± 4.3 ab	44.0 ± 2.8 ab	43.3 ± 3.1 ab	38.3 ± 1.5 a	44.9 ± 1.1 ab	44.6 ± 3.3 ab
hexyl acetate	EA2	12.6 ± 1.9 b	13.8 ± 0.8 ab	11.2 ± 1.5 ab	11.1 ± 1.3 ab	11.2 ± 0.8 ab	13.6 ± 1.2 b	9.6 ± 0.4 a	10.8 ± 0.3 ab	11.5 ± 1.0 ab
2-phenylethyl acetate	EA3	2.6 ± 0.2 c	2.5 ± 0.1 bd	1.6 ± 0.4 a	2.2 ± 0.2 bc	2.4 ± 0.2 bc	2.0 ± 0.2 ab	1.5 ± 0.1 a	2.3 ± 0.1 bc	2.3 ± 0.2 bc

(continue)

Table A4. *(continue)*

diethyl succinate	EI1	25.2 ± 1.3 d	23.6 ± 2.2 cd	16.3 ± 3.1 ab	20.0 ± 2.4 abcd	21.7 ± 1.8 cd	19.5 ± 1.5 abc	15.5 ± 0.6 a	20.8 ± 0.9 bcd	21.2 ± 1.0 bcd
diethyl malate	EI2	0.9 ± 0.2 abc	1.2 ± 0.2 bc	0.9 ± 0.1 abc	0.8 ± 0.3 abc	0.5 ± 0.0 a	0.6 ± 0.3 a	0.8 ± 0.2 abc	1.4 ± 0.3 c	0.7 ± 0.1 ab
ethyl hydrogen succinate	EI3	2.2 ± 0.6 bc	3.1 ± 0.2 bc	0.4 ± 0.1 a	2.3 ± 0.5 bc	2.5 ± 0.2 bc	2.0 ± 0.5 b	2.3 ± 0.7 bc	3.5 ± 0.6 c	2.2 ± 0.6 bc
3-methylbutyl octanoate	AE	1.2 ± 0.1 a	2.1 ± 0.1 ab	1.4 ± 0.9 a	1.7 ± 0.1 ab	2.4 ± 0.6 ab	2.9 ± 0.8 b	1.6 ± 0.3 ab	1.3 ± 0.2 a	2.4 ± 0.4 ab
γ-butyrolactone	L	0.8 ± 0.1	0.8 ± 0.1	0.7 ± 0.1	0.8 ± 0.0	0.8 ± 0.0	0.7 ± 0.1	0.7 ± 0.1	0.7 ± 0.1	0.8 ± 0.0
methionol	S	0.3 ± 0.0 bc	0.3 ± 0.0 bc	0.2 ± 0.1 b	0.2 ± 0.1 bc	0.3 ± 0.0 c	0.2 ± 0.0 bc	0.0 ± 0.0 a	0.2 ± 0.1 bc	0.3 ± 0.1 c
linalool	T1	0.7 ± 0.1 bc	0.8 ± 0.1 c	0.6 ± 0.1 ab	0.7 ± 0.1 bc	0.8 ± 0.0 c	0.8 ± 0.1 c	0.5 ± 0.0 a	0.7 ± 0.1 abc	0.8 ± 0.1 c
α-terpineol	T2	1.2 ± 0.2 c	0.9 ± 0.1 bc	0.5 ± 0.2 a	0.7 ± 0.1 ab	0.8 ± 0.1 b	0.8 ± 0.1 ab	0.5 ± 0.0 a	0.7 ± 0.1 ab	0.9 ± 0.1 b

AC: acids; AL: alcohols; D: diols; EE: ethyl esters; EA: acetate esters; EI: aging esters; AE: other esters; L: lactones; S: sulfur compounds; T: terpenes.
